# Research on order batching optimization based on improved NSGA-II algorithm

**DOI:** 10.1371/journal.pone.0319182

**Published:** 2025-02-25

**Authors:** Huiyue Xu, Juping Shao, Yanan Sun

**Affiliations:** 1 School of Business, Suzhou University of Science and Technology, Suzhou, Jiangsu, China; 2 Suzhou Youlesai Supply Chain Management Co., Ltd., Suzhou, Jiangsu, China; Indian Institute of Technology Kanpur, INDIA

## Abstract

In the context of e-commerce, the order batching optimization problem in e-commerce warehousing centers has been addressed by establishing a model aimed at minimizing the order picking time, order delay costs, and picking costs, as well as achieving workload balance. An improved NSGA-II algorithm has been designed, which enhances the search capability and solution diversity by introducing new selection mechanisms and crossover mutation strategies. This approach more effectively balances multiple optimization objectives and validates the effectiveness of the model and algorithm with case studies, while also conducting sensitivity analysis on model parameters. The research results indicate that the established model and the designed algorithm are effective, providing a theoretical basis and practical significance for the optimization of order picking efficiency in e-commerce distribution centers.

## Introduction

The rapid development of the e-commerce industry has led to high-frequency, small-batch, and multi-variety logistics distribution demands, making the efficiency of the order picking process particularly crucial. In large e-commerce warehouses, the picking operation accounts for 30%-40% of the total working time and up to 60% of the costs [1]. As consumers’ demands for logistics service quality increase, the timely delivery of orders has become a focal point of customer attention. Failure to complete order picking and shipping within the specified time can lead to decreased customer satisfaction, increased return rates, more customer complaints, and even damage to the brand image [2]. Therefore, e-commerce warehouses need to find a balance between improving picking efficiency and meeting order delivery deadlines [3].

In actual operations, the efficient integration of similar orders and the planning of work routes are crucial for enhancing picking efficiency [4]. Research both domestically and internationally generally agrees that reasonable order batching is an effective method to improve picking efficiency [5–7]. Moreover, actual picking work is usually carried out by multiple pickers working in coordination. However, in actual production, due to the randomness of orders and the uncertainty of order volume,there is often a phenomenon where a single picker is assigned a large number of tasks. Such an unbalanced arrangement not only causes fatigue and dissatisfaction among overburdened employees, reducing their motivation to work, but also affects the overall picking efficiency [8,9]. Therefore, when optimizing the picking process, the workload balance of picking workers should also be considered.

However, existing studies have not considered the combination of picking efficiency and reducing order delays. Most studies assume that there is only one picker, which does not align with the reality of e-commerce warehouses, and the workload balance of staff after order batching also needs to be considered. Therefore, this paper focuses on the order picking problem and studies a model that considers order batching, designing an improved NSGA-II algorithm. By introducing new selection mechanisms and crossover mutation strategies, the search capability and solution diversity are enhanced, thus more effectively balancing multiple optimization objectives.

The rest of this paper is organized as follows: Section 2 provides a literature review on the order batching problem, Section 3 describes the problem and presents a new mathematical model formulation, Section 4 introduces the NSGA-II algorithm and improves the traditional crossover, mutation, and selection methods by introducing a disturbance factor, as well as proposes an improved fast non-dominated sorting algorithm based on the divide-and-conquer approach. Additionally, a grid-based crowding distance calculation method is introduced to enhance algorithm performance. Section 5 evaluates the algorithm’s performance and the effect of solving sub-problems simultaneously through numerical experiments. Section 6 summarizes the research findings. Section 7 discusses the practical applications and impacts of the research in warehouse management. Section 8 discusses the limitations and potential challenges in real-world implementation. Finally, the paper concludes with prospects for further research.

## Literature review

Existing research on order batch optimization primarily focuses on how to effectively enhance the efficiency of goods picking and reduce delays in the picking process. In the study of improving the efficiency of goods picking, Chengyu Cheng et al. [10] proposed a mathematical model that maximizes order similarity based on the similarity of order items. Magdalena Matusiak [11] constructed an order batch optimization problem aimed at minimizing the picking route and provided a method for merging order batches based on channel similarity. Ehsan Ardjmand et al. [12] developed an order batch optimization model based on the shortest completion time, utilizing a hybrid simulation annealing and ant colony algorithm to solve the clustering problem of order batch optimization. Li Shizhen and Du Wenhong [13] transformed the order batching problem into a clustering problem with the shortest picking route as the goal and solved it using heuristic algorithms. Huang [14] established an order batching optimization model aimed at minimizing picking time and proposed an order batching model based on channel similarity. Ardjmand et al. [15] established an order batching optimization model aimed at minimizing the total completion time of orders and solved it using a hybrid parallel simulated annealing and ant colony optimization algorithm. Menendez et al. [16] aimed to minimize the completion time of orders and solved it using variable neighborhood descent and variable neighborhood search algorithms. Aboelfotoh et al. [17] proposed a heuristic algorithm for batch sorting and compared and analyzed its solution performance. Gil Borrás et al. [18] studied the order batching problem in online order systems, proposed a multi-stage variable neighborhood descent metaheuristic algorithm, constructed mathematical models for order batching aimed at minimizing order execution time, minimizing order picking time, and minimizing workload imbalance, and solved the models, verifying the effectiveness of the algorithms with examples. Jiang et al. [19] proposed the Situation-based Seed (SBS) algorithm, aiming to minimize the completion time of all orders, and solved the mathematical model of order batching and scheduling sequence optimization problems. Pérez-Rodríguez et al. [20] aimed to minimize the fulfillment time of all customer orders, studied the online order batching problem, and proposed a continuous estimation method based on distribution algorithms for solving the problem, which significantly reduced the average order fulfillment time. Zhang et al. [21] addressed the online order batching problem with the goal of minimizing turnover time, also known as the maximum completion time of all batches (i.e., the time required to collect all orders, including waiting time, path time, batch processing, and service time). The aforementioned studies mainly focused on optimizing picking efficiency and did not consider customer satisfaction constraints, which could lead to order delays and a significant decrease in customer satisfaction during order batch picking.

In the research on reducing delays in goods picking, André Scholz et al. [22] proposed a variable neighborhood-based order batch optimization method. Ri-Huan Huang et al. [23] constructed a mathematical model for order merging, sorting, and path optimization, and optimized it using a combined ant optimization and genetic algorithm. Feng Ailan et al. [24] presented a multi-objective order batch problem with the fewest delayed orders and the shortest average delay time, solved using the VNS algorithm. Shuanglu Zhang et al. [3] discussed an order batching and personnel scheduling optimization problem, aiming to minimize total costs, and the analysis showed that reasonable order and personnel allocation would enhance the picking efficiency of the warehouse system. Xuan Yang et al. [25] studied a multi-variety picking strategy based on HGA-VNS, focusing on batch sorting problems based on minimizing order delays under conditions such as off-season/peak season, regional effects, and item configuration strategies. Tsai et al. [26] constructed an order batching optimization model aimed at minimizing the sum of delayed completion penalty costs and solved it using a multi-stage genetic algorithm. Azadnia et al. [27] considered the completion deadlines of orders, constructed a weighted association rule algorithm to calculate the association between orders, and built an order batching optimization model that maximizes the association between orders, solved using a genetic algorithm. Henn and Schmid [28] demonstrated how to use metaheuristic algorithms to minimize the total delay time of a given set of customer orders with a given scheduled date. The aforementioned order batching problems did not mention the similarity between orders; if the number of orders is large, the complexity of the solution algorithm will also greatly increase. Moreover, most studies assume that there is only one picker, which does not match the actual warehouse picking environment.

## Problem description and model establishment

### Problem description and assumptions

**Fig 1:**
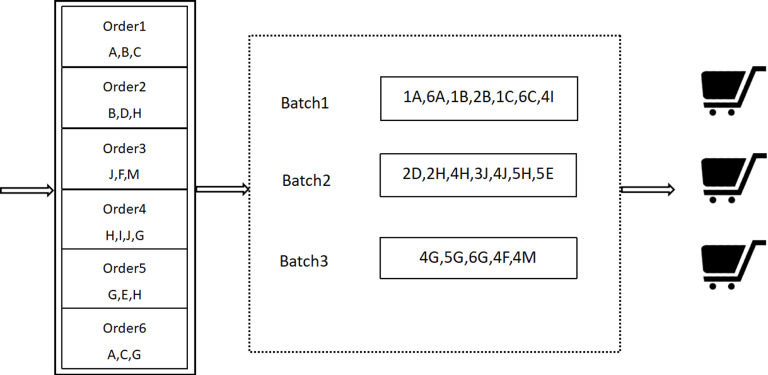
Order Batching and Picking Process.

In the e-commerce environment, the order picking problem consists of three components: order arrival, order batching, and picking routing [29]. The order picking problem can be described as follows: within a certain time period, a warehouse receives multiple customer orders, each containing information such as the types of goods, quantities, and deadlines. These orders are grouped into different batches based on certain rules (such as item similarity between orders, deadlines, proximity of storage locations, etc.). Then, pickers perform the picking operations based on these batches. The specific process is shown in Fig 1.

**Fig 2:**
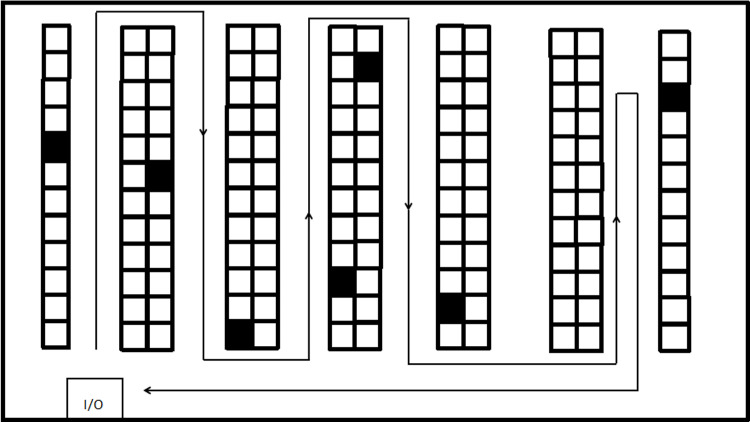
S-Shaped Strategy.

This study takes the single-zone warehouse [21] as an example, where pickers follow an S-shaped route for picking. That is, the picker enters from one end of the aisle containing the items to be picked and exits from the other end. During the picking process, the picker only needs to enter aisles that contain the required items, and can skip aisles that do not contain any items to be picked. The picking process is shown in Fig 2. When the picker receives a batch picking task, the start time of this batch is recorded. Upon completion of the task and return to the starting point, the batch is considered finished. The delay of an order refers to the difference between the actual completion time of the order and its picking deadline.

To establish a mathematical model for order batching and batch allocation optimization, the following assumptions are made:

1) Picking staff must complete the current batch of tasks before starting the next one, and tasks cannot be interrupted during execution.
2) The total product volume in a batch of orders does not exceed the capacity limit of the picking cart.
3) In this warehouse, the storage location of goods is fixed, with one item stored at each location. Picking staff moves at a constant speed within the distribution center, walking in the middle of the aisles while picking items from both the left and right sides.
4) There are no stockout issues during the picking process.
5) Emergency order insertion is not considered.

### Order batching and allocation optimization model

The definitions of the model parameters and decision variables are presented in Table 1.

**Table 1:** Presents the definitions of model parameters and decision variables.

Symbol	Explanation
*A*	Set of orders, $a\in A=\left\{1,2,...,|A|\right\}$
*B*	Set of batches, $b,b^{'}\in B=\left\{1,2,...,|B|\right\}$
*Z*	Set of pickers, $z\in Z=\left\{1,2,...,|Z|\right\}$
*Q*	Picker set, $j,k,q\in Q=\left\{1,2,...,|Q|\right\}$
*V*	Maximum cargo volume that picking equipment can accommodate
$V_{a}$	The total volume of goods contained in order *a*
*v*	The walking speed of the picker
$v_{\text{p}}$	The picking speed of the picker
*c*	The cost of picking each item from the shelf
$t_{a}$	The actual delivery date of order *a*
$t_{b\text{,end}}$	The completion time of batch *b*
$t_{b,\text{ser}}$	The service time required for batch *b*
$t_{a\text{,e}}$	The critical time when the delivery deadline reaches the penalty threshold
$t_{a,\text{me}}$	The critical time when the delivery deadline reaches the maximum penalty threshold
*h*	The distance between two adjacent aisles in the warehouse.
*l*	The length of the aisles in the warehouse
$\theta_{a}$	The number of items included in order *a*
$M_{b}$	The number of item types included in batch *b*
$\eta_{b,j},\eta_{b,k}$	The sequence order of items *j* and *k* in their respective locations during the picking of batch *b*
$d_{b}$	The walking distance required to pick the items in batch *b*
$n_{b}$	The number of aisles that need to be accessed in picking batch *b*
$n_{b,\text{l}}$	The aisle number of the leftmost aisle that needs to be accessed during the picking process.
$n_{b,\text{r}}$	The aisle number of the rightmost aisle that needs to be accessed during the picking process
$D_{b,\text{r}}$	The distance from the aisle entrance to the farthest picking item when picking the items in the rightmost aisle of batch *b*
$S(t_{a})$	The penalty function for the delay time of order *a*
$g_{b,j,k}$	1, if leaving location *j* to go to location *k*;0, otherwise
$x_{a,b}$	1,if order *a* belongs to batch *b*;0, otherwise
$y_{z,b,b^{'}}$	1,if batch $b^{'}$ is picked immediately after batch *b* by picker *z*; 0,otherwise
$p_{b,z}$	1,if batch *b* is assigned to the z−th picker;0,otherwise

(1)The penalty function $S(t_{a})$ for any order *a* is determined by the required completion time and the actual completion time of the order. The difference between the actual completion time and the required completion time corresponds to a specific time penalty function.


$$S(t_{a})\left\{\begin{array}{l} 0,t_{a}<t_{a\text{,e}}\\ \alpha (t_{a}-t_{a\text{,e}}),t_{a\text{,e}}<t_{a}<t_{a,\text{me}}\\ \beta (t_{a}-t_{a,\text{me}}),t_{a,\text{me}}<t_{a} \end{array}\right\}$$ (1)

**Fig 3:**
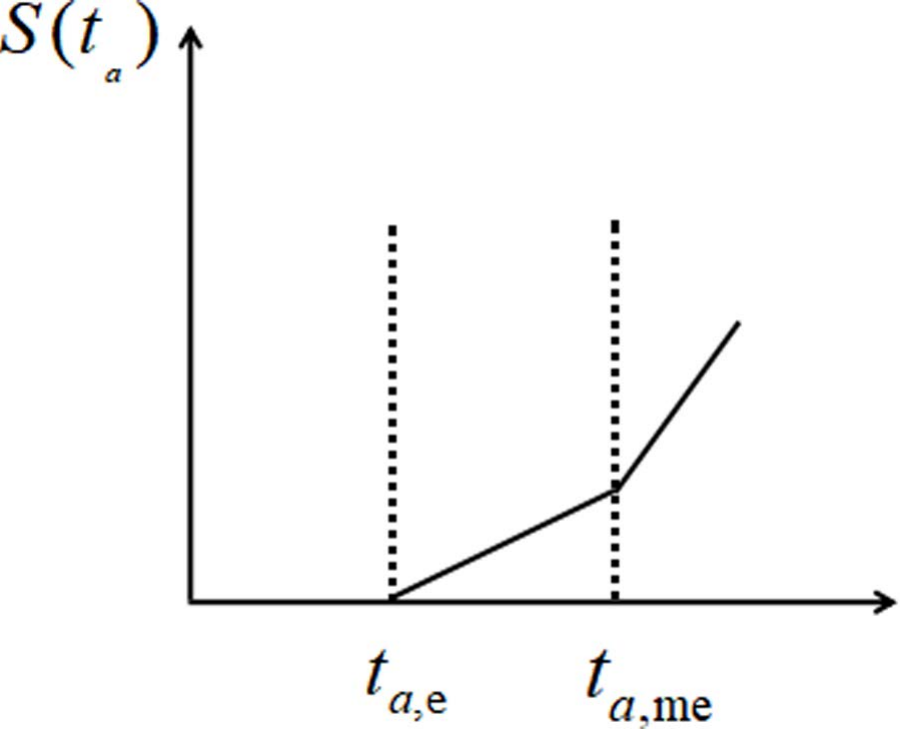
Changes in Punishment Function.

The penalty function is a piecewise function. When the actual delivery time of order a is less than the critical time at which penalties begin to apply, there is no penalty value. When the actual delivery time of order a falls within $\left[t_{a\text{,e}},t_{a,\text{me}}\right]$, the penalty function increases with the duration of the delay, where α is the penalty coefficient for that interval. When the actual delivery time of order a exceeds the critical time for the maximum penalty, the penalty function also increases with the delay time, with β as the penalty coefficient for that interval. The changes in the penalty function related to order delay time are illustrated in Fig 3.

Based on the above analysis, the following order batching optimization model is established, with the objectives of minimizing order picking time, total order delay cost, and picking cost, as well as minimizing the maximum difference between the picking time of the most utilized picker and the average picking time of all pickers in the system:


$$\min f_{1}=\sum_{b\in B}t_{b\text{,end}}$$ (2)

$$\min f_{2}=\sum_{a\in A}S(t_{a})+\sum_{b\in B}\sum_{a\in A}c\theta_{a}x_{a,b}$$ (3)

$$\min f_{3}=\min\left[\max\sum_{b\in B}p_{b,z}t_{b,\text{ser}}-\sum_{k\in K}\frac{t_{b,\text{ser}}}{z}\right]$$ (4)

s.t.


$$\sum_{b\in B}x_{a,b}=1,\forall a\in A$$ (5)

$$\sum_{a\in A}V_{a}x_{a,b}\leq V,\forall b\in B$$ (6)

$$\eta_{b,j}-\eta_{b,k}+M_{b}g_{b,j,k}\leq M_{b}-1$$ (7)

$$\sum_{j\in M}g_{b,j,k}=1,\forall k\in M,j\neq k$$ (8)

$$\sum_{k\in M}g_{b,j,k}=1,\forall j\in M,j\neq k$$ (9)

$$\sum_{b\in B\left\{0\right\}}\sum_{z\in Z}y_{z,b,b^{'}}=1,\forall z\in Z,\forall b\in B$$ (10)

∑b'∈Byz,b,b'≤1,∀z∈Z,∀b∈B (11)

$$t_{b^{'},\text{end}}=�\sum_{\text{z}\in Z}\sum_{b\in B\cup \left\{0\right\}}t_{b\text{,end}}+t_{b^{’},\text{ser}}�y_{z,b,b^{'}},\forall b^{'}\in B$$ (12)

$$t_{b,\text{ser}}=\frac{d_{b}}{v}+x_{a,b}\theta_{a}v_{\text{p}},\forall b\in B,\forall a\in A$$ (13)

$$d_{b}=\left\{\begin{array}{l} (n_{b,\text{l}}-1)h+n_{b}l+(n_{b}-1)h+(n_{b,\text{r}}-1)h,n_{b}isanevennumber.\\ (n_{b,\text{l}}-1)h+(n_{b}-1)l+2D_{b,\text{r}}+(n_{b}-1)h+(n_{b,\text{r}}-1)h,n_{b}isanoddnumber.\\ (n_{b,\text{l}}-1)h+2D_{b,\text{r}}+(n_{b,\text{r}}-1)h,n_{b}=1,n_{b,\text{l}}=n_{b,\text{r}}=0. \end{array}\right\}$$ (14)

Eqs (2–4) represent the optimization objectives. Eq (2) minimizes the order picking time. Eq (3) minimizes the order delay cost and picking cost. Eq (4) minimizes the maximum difference between the picking time of the busiest picker and the average picking time of all pickers. Constraint (5) ensures that each order can only be assigned to one batch, and no order can be split across multiple batches. Constraint (6) specifies that the total volume of goods in each batch must not exceed the maximum capacity of the picking device. Constraint (7) ensures that there are no sub-tours in the picking routes for each batch. Constraints (8 and 9) ensure that any item can only belong to one batch and be picked only once. Constraint (10) ensures that each batch is assigned to one picker. Constraint (11) ensures that, for any picker, there is at most one subsequent batch assigned after completing a batch. Constraint (12) defines the end time of the picking operation for each batch. Constraint (13) specifies that the batch service time consists of two components: the walking time and the item retrieval time for the picker. Constraint (14) defines the walking distance required for picking each batch.

## Algorithm design

### Overview of the NSGA-II algorithm

This paper constructs a multi-objective optimization model aimed at minimizing picking time, order delay costs, and picking costs, while simultaneously reducing the gap between the maximum picking time of the pickers and the overall average picking time. However, there are inherent conflicts between these objectives, meaning that improving one objective will inevitably lead to the deterioration of at least one other objective. Additionally, the order batching problem has been proven to be a difficult problem [30], with exact solution algorithms facing computational challenges, and the computation time increasing exponentially with problem scale. Currently, metaheuristic algorithms are often used to solve such complex problems.

The Non-Dominated Sorting Genetic Algorithm (NSGA) was proposed by Deb in 2000. Building on this, Deb and Pratap introduced the NSGA-II algorithm in 2002 [31], which utilizes a fast non-dominated sorting method to reduce algorithm complexity. It incorporates a crowding distance and a crowding comparison algorithm, resulting in a more uniform distribution of Pareto solutions and increased population diversity. The introduction of an elitism strategy expands the sampling space, leading to faster application, lower complexity, and better uniformity of the solution set, making it widely applicable in various domains.

## Improved NSGA-II algorithm solution

### Chromosome encoding and population size design

Genetic algorithms can utilize various encoding methods, including binary encoding, Gray code encoding, real number encoding, and permutation encoding. While all these methods achieve similar computational precision, binary encoding is the most commonly used. However, with many design variables, the encoding and decoding processes can become complex. In contrast, real number encoding offers certain advantages and appears more natural. Therefore, this paper adopts real number encoding.

### Fast non-dominated sorting

After initializing the population, two parameters, $\delta_{\varepsilon }$ and $\mu_{\varepsilon }$,are set for each individual *ε* in the population.Here $\delta_{\varepsilon }$ represents the set of individuals dominated by p,while $\mu_{\varepsilon }$ indicates the number of individuals dominating *ε*. The specific steps are as follows:

Step 1: Calculate the dominance set $\delta_{\varepsilon }$ and domination count $\mu_{\varepsilon }$,for each individual, setting the layer number w=1.

Step 2: Place all non-dominated solutions ($\mu_{\varepsilon }=0$) into the first layer, marking their rank as $r_{w}=1$.

Step 3: For each individual in the set $\delta_{\varepsilon }$,decrement $\mu_{\varepsilon }$ by 1 and assign them to the next rank $r_{w}+1$.

Step 4: Repeat Steps 1 to 3 until all parent individuals in the population are sorted into layers.

To further optimize the efficiency of non-dominated sorting, this paper adopts an improved fast non-dominated sorting algorithm based on the divide-and-conquer approach. In the traditional non-dominated sorting algorithm, the computational complexity is O(MN²) (where M is the number of objectives and N is the population size), which is inefficient for large-scale populations. By implementing the following optimization strategies, the complexity is reduced to O(MN log N):

Divide Phase: The population is first sorted based on the first objective, which is then used to partition the population into several smaller sub-populations.

Conquer Phase: Non-dominated sorting is recursively performed within each sub-population. During this phase, the dominance relationships are only compared among individuals within the same sub-population, avoiding global comparisons.

Combine Phase: The sorted non-dominated sets from the sub-populations are merged into a global non-dominated set. During the merging process, only the boundary solutions across sub-populations are compared for dominance, further reducing unnecessary comparisons.

### Crowding distance calculation

To address the high computational complexity of crowding distance calculation in traditional NSGA-II, this paper integrates an improved fast non-dominated sorting mechanism and introduces a grid-based crowding distance calculation method to reduce redundant comparisons between individuals. The grid-based method is implemented through the following steps:

Grid Partitioning of the Objective Space: The objective space is divided into evenly spaced grid cells, and the population is mapped to the corresponding grid cells.

Fast Crowding Distance Update: The density of individuals within each grid cell is calculated, and the crowding distance is updated using information from adjacent grid cells. This approach avoids the high computational cost of pairwise comparisons in traditional methods, significantly improving computational efficiency.

The improved crowding distance calculation formula is as follows:


$$s_{i}=\sum_{u=1}^{U}\frac{f_{u,i+1}-f_{u,i}}{f_{u,\max}-f_{u,\min}}$$

$f_{k,\max},f_{k,\min}$ represents the maximum and minimum values of the k-th objective function, respectively.

### Design of selection, crossover, and mutation operators

Commonly used selection operators include roulette wheel, tournament, and truncation selection. In this paper, the tournament selection method is employed, requiring the definition of two parameters: the population and competing individuals. From n individuals, n/2 are randomly selected. Quality individuals are chosen based on their rank and crowding distance, where the rank is compared first, and if ranks are identical, crowding distance is considered next. This method has low computational complexity, is easy to parallelize, and selects individuals with better fitness, making it less prone to local optima without the need to analyze all fitness values.

**Fig 4:**
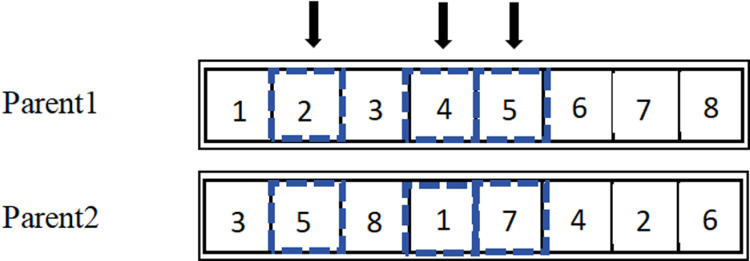
Steps of PBX Crossover.

**Fig 5:**
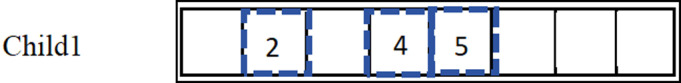
Steps of PBX Crossover.

**Fig 6:**
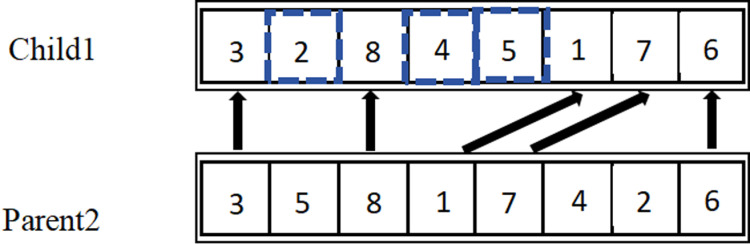
Steps of PBX Crossover.

**Fig 7:**
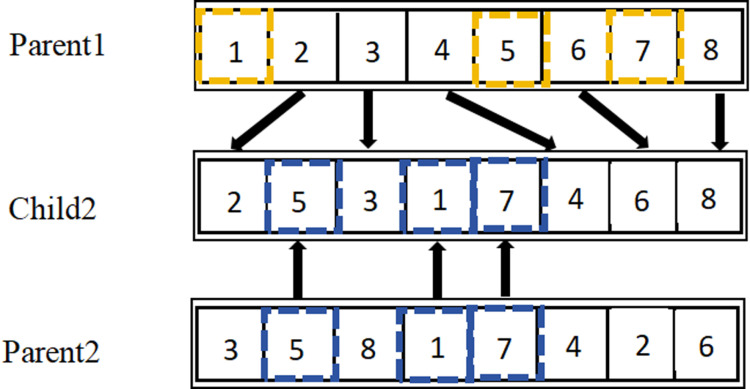
Steps of PBX Crossover.

Crossover operations involve exchanging segments of chromosomes in a specific manner to generate two entirely new individuals.A random number *o* is generated from the interval (0,1), which is then compared with the crossover probability *ρ*,typically ranging from 0.4 to 0.99.If *o* ≤ *ρ*,crossover operations are performed;if *o*> *ρ*,the two parent individuals are reintegrated into the next generation of the population. In conjunction with the natural number encoding method, this paper selects Position-Based Crossover (PBX) suitable for discrete optimization problems. The main steps of PBX are as follows:

(1) Randomly select several genes from a pair of chromosomes P1 and P2 in the parent population. The selected positions can be non-contiguous, but they must be the same in both chromosomes, as shown in Fig 4.

(2) Generate offspring C1 by copying the selected genes from P1 into C1 according to their positions, as shown in Fig 5.

(3) Remove the genes selected from P1 in P2,and fill the remaining parts in order into C1. The result is shown in Fig 6.

(4) C2 retains the genes from P2 at the same positions. After removing the selected genes from P1, the remaining genes are filled into C2 in order. The resulting chromosome C2 is the new chromosome obtained from the crossover operator, as shown in Fig 7.

**Fig 8:**
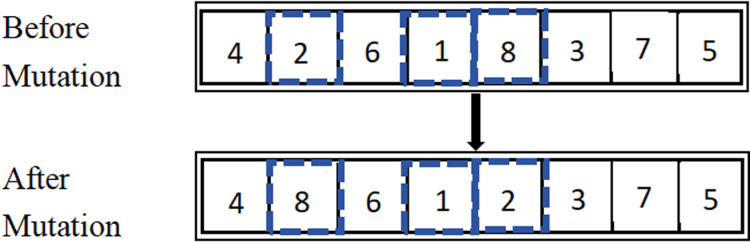
Steps of PBX Crossover.

Mutation operations can significantly enhance the local search capability of genetic algorithms. In this study, we employ Swap Mutation, where each individual in the population after crossover is subjected to mutation according to a specified mutation probability. This is primarily achieved by randomly generating two distinct mutation points on the individual and exchanging the genes at these loci to create new individuals. The advantage of this approach is that the mutation process does not generate new genes; it preserves high-quality gene individuals, preventing their loss, and also avoids the generation of duplicate solutions during the iterative process. The Swap Mutation process is illustrated in Fig 8.

To enhance the convergence of the algorithm, an adaptive scaling factor λ is introduced. This factor allows for a larger number of selected gene loci in the early iterations of the algorithm, which gradually decreases in the later stages.


$$\lambda =0.8+0.5\frac{G_{\max}-G_{\partial }}{G_{\max}}$$

**Fig 9:**
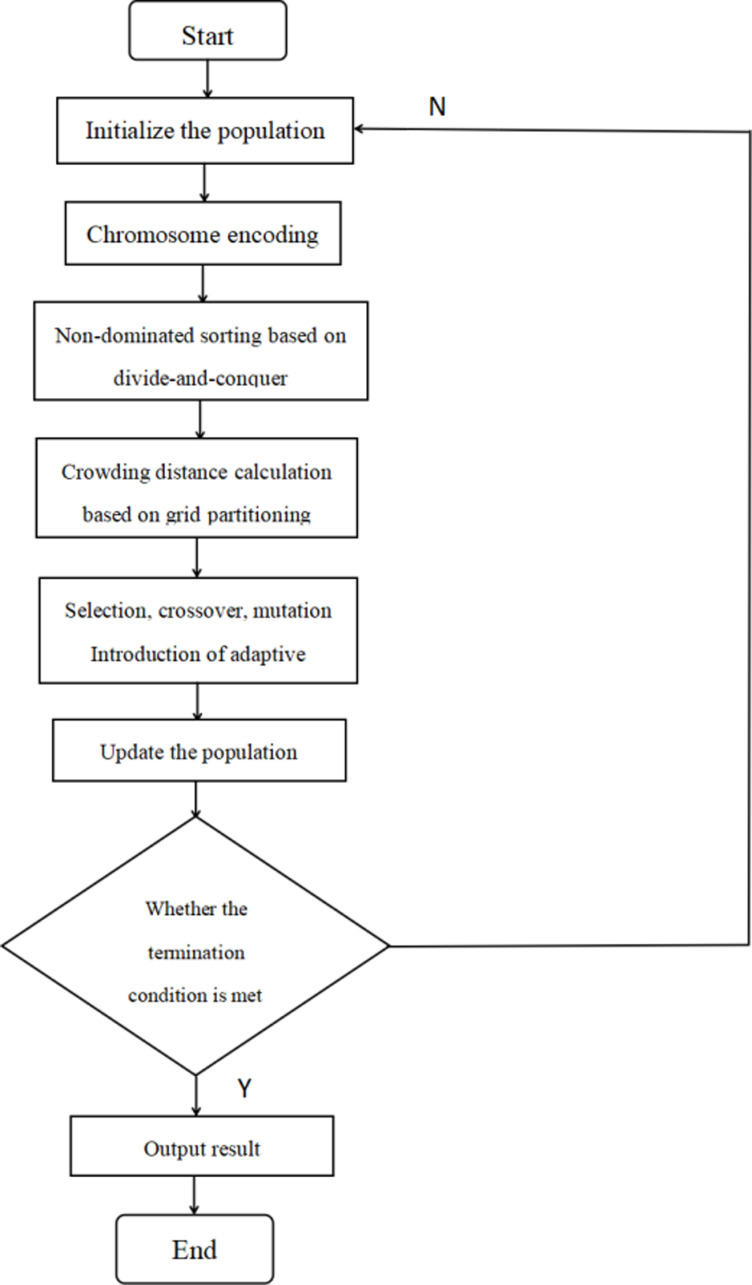
Specific Process of the Improved NSGA-II Algorithm.

Here, $G_{\max}$ represents the maximum number of iterations,and  ∂  denotes the ∂−th iteration. As the number of iterations increases, this allows the crossover operator to select more gene loci in the early stages of iteration while reducing the number of selected loci in the later stages. The specific process of the improved NSGA-II algorithm is illustrated as follows Fig 9:

## Example verification

### Case description

**Fig 10:**
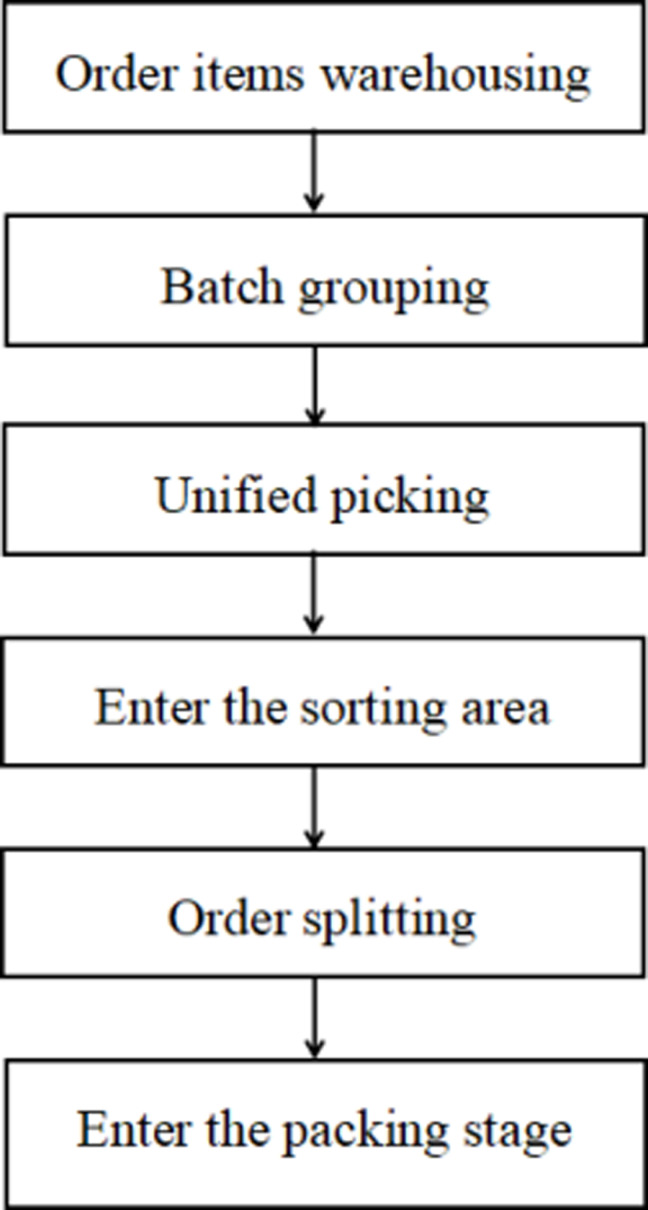
The specific process of warehouse operations.

The primary data for this paper is sourced from the actual reports of a company’s distribution center. To ensure the comprehensiveness and accuracy of the data, recent research reports published by the company were reviewed, covering detailed information about its Warehouse Management System (WMS) and picking processes. Additionally, the study further explored the picking methods employed by the center and their operational details. The company’s picking methods are as follows:Order Grouping: The Warehouse Management System groups order items into batches to optimize the allocation of picking tasks.Unified Picking: Items are picked based on batch lists, and the goods are delivered to the sorting area upon completion.Order Splitting: In the sorting area, orders are manually split and organized according to customer requirements.Order Packing: After order splitting is completed, the goods are sent to the packing area for final packaging and shipment preparation.The detailed process is illustrated in the following diagram Fig 10.

This paper analyzes actual order data collected over a specific period. The number of product types in each order follows a uniform distribution on [1,5], and the quantity of each product follows a uniform distribution on [1,8]. The warehouse has a single-zone layout with 120 storage locations and two cross-aisles, and the aisle layout is shown in Fig 2. The starting point (I/O) of the warehouse is located in the bottom-left corner. There are 10 aisles in total, numbered from 1 to 10 from left to right. Each aisle has 12 storage locations on both sides, and each location holds only one type of product.

The distance between adjacent storage locations is 2 m, the aisle length is 12 m, and the distance between adjacent aisles is 1 m. The maximum picking capacity of the picking equipment is 30 units. The picking personnel have a walking speed of 1.5 m/s, and the speed of extracting items is 1.0 m/s. The picking cost is 0.7 yuan per item. An S-shaped picking route is adopted for the picking process.

To evaluate the effectiveness of the optimization strategy, order data of different scales were analyzed as follows:Small-scale: Orders from April 13 to April 14 were analyzed.Medium-scale: Orders from April 15 to April 17 were analyzed.Large-scale: Orders from April 18 to April 22 were analyzed.

### Case results

The following calculations were performed on a personal office computer with 8GB of memory and a 1.1GHz dual-core Intel Core i3 processor. The programming platform used was PyCharm CE 2022, and an improved NSGA-II algorithm was employed to solve the order batching problem. The model is based on three objective functions: minimizing picking time, minimizing order delay costs, and minimizing the maximum difference between the picking time of the worker with the highest picking time and the average picking time of all workers in the system. The solving results for orders of different scales are shown in Tables 2–4. As shown in Table 2, the small-scale order was divided into 5 batches.

**Table 2:** Results of Small-Scale Cases.

Batch	Order Number
1	11-1 62-1 76-1 32-1 93-1 24-1 3-1 20-1 13-1 22-1 63-1 52-1 48-1 99-1 27-1 14-1 96-1 61-1 70-3 42-1 90-1 75-1 59-1
2	55-1 54-1 88-1 89-1 65-1 66-2 26-1 39-1 68-1 58-1 16-1 84-1 94-1 69-1 29-1 64-2
3	82-1 44-1 63-2 97-1 100-1 8-1 30-1 60-1 29-2 74-1 25-1 60-2 334-1 12-1 77-1 41-1 51-1 56-1 10-1 67-1 78-1 40-1 1-1 69-2 61-2
4	81-1 31-1 95-1 87-1 62-2 21-1 66-1 19-1 86-1 18-1 57-1 45-1 2-1 9-1 72-1 70-2 23-1 92-2 5-1 36-1 49-1 85-1 71-1 28-1 35-1 73-1 33-1
5	17-1 7-1 71-2 37-1 67-2 68-2 46-1 83-1 98-1 70-1 64-1 50-1 4-1 47-1 6-1 53-1 80-1 15-1 43-1 79-1 92-1 91-1 38-1

**Table 3:** Results of Medium-Scale Cases.

Batch	Order Number
1	177-1 29-1 146-1 31-1 146-2 33-1 196-1 120-2 140-1 79-1 90-1 171-1 19-2 16-1193-1 77-1 148-1 121-1 143-2 54-1 130-2 102-1 113-1 80-1 90-2 146-2 1-1 126-2 22-1 112-1 56-1 137-2 149-1 125-1 136-1 188-1 178-1 145-2 195-1 160-1 174-1 46-1 123-1 13-1
2	129-2 175-1 49-1 105-1 9-1 170-1 143-1 47-1 68-1 144-2 118-1 111-1 37-1 103-1 94-1 139-1 10-2 127-2 25-1 60-1 142-1 138-2 14-1 21-1 4-1 173-2 11-1 194-1 23-1 168-1 89-1 86-1 106-1 181-1 28-1 192-1 197-1 15201 109-1 147-1 108-1 6-1 58-2 7-1 42-1 190-1 159-1 141-2 155-1 189-1 18-1 134-1 128-2 118-2
3	24-1 191-1 169-2 164-1 134-2 81-1 88-1 163-1 44-1 69-1 145-1 55-1 38-1 84-1 2-1 58-1 166-1 5-1 87-1 19-1 120-1 63-1 110-1 101-1 95-1 176-1 64-1 71-1 187-1 197-2 138-1 107-1 135-2 104-1 117-1 179-1 133-2 12-1 51-1 132-1199-1 182-1 27-1 70-1 133-1 83-1 78-1 17-1 26-1
4	l32-1 30-1 50-1 34-1 97-1 67-1 73-1 66-1 74-1 142-2 53-1 15-1 35-1 165-1 153-1 48-1 129-1 135-1 161-1 150-1 183-1 57-1 85-1 124-2 122-1 184-1 132-2 91-2 130-1 65-1 115-1 127-1 123-2 137-1 172-1 119-2 82-1 10-1 43-1 96-1 122-2 156-1 36-1 158-1 125-2 139-2 131-1 169-1 99-1 151-1 141-4 76-1 91-1 61-1 200-1 75-1
5	185-1 119-1 186-1 8-1 116-1 100-1 176-2 124-1 52-1 20-1 173-1 2-2 40-1 136-2 3-2 157-1 141-1 162-1 3-1 98-1 59-1 121-2 128-1 114-1 198-1 41-1 131-2 93-1 141-3 45-1 180-1 92-1 39-1 144-1 154-1 126-1 62-1 62-1 72-1 167-1

**Table 4:** Results of Large-scale Cases.

Batch	Order Number
1	242-1 191-1 241-1 260-2 247-2 254-2 210-1 115-1 268-2 133-1 219-2 278-1 74-1 28-1 108-1 99-1 199-1 40-1 218-2 177-1 16-1 105-1 55-1 193-1 142-1 65-1 45-1 135-1 279-2 261-1 36-1 29-1 213-2 162-1 12-1 226-2 267-1 209-1 227-2 187-1 257-2 192-1 295-1 211-1 87-1 61-1 24-1 241-2 274-2 300-2 54-1 254-1 271-1 171-1 246-1 273-3 185-2 78-1 247-1 147-1 63-1 227-1 216-1 156-1 107-2 161-1 277-2 122-1 25-1 169-1 35-1 248-2 56-1 273-2 259-2 129-1 190-1 144-1 138-1
2	256-1 157-1 51-1 225-1 46-1 239-2 160-1 149-1 230-2 139-1 269-2 100-1 234-1 276-2 163-1 210-2 300-1 236-1 261-2 117-1 218-1 245-2 226-1 113-1 183-1 239-1 91-1 102-1 287-1 286-1 73-1 149-2 77-1 90-1 175-1 248-1 104-1 27-1 111-1 224-1 203-1 19-1 264-2 182-1 174-1 279-1 7-1 206-1 220-1 49-1 229-2 136-1 204-1 293-1 291-1 232-1 275-1 119-1 185-1 191-2 69-1 215-2 148-1 43-1 234-2 292-1 235-2 165-1 273-1 96-1 238-1
3	158-1 6-1 103-1 217-2 271-1 269-1 297-1 266-1 233-2 194-1 290-1 257-1 130-1 207-2 272-1 198-1 272-2 251-2 41-1 93-1 9-1 205-1 196-1 107-1 84-1 263-2 154-1 146-1 258-2 252-2 243-2 243-1 152-1 268-2 178-1 153-1 14-1 282-1 179-1 110-1 237-1 89-1 299-1 281-1 20-1 3-1 221-2 211-2 10-1 88-1 85-1 70-1 277-1 245-1 289-1 125-1 140-1 246-2 26-1 134-1 52-1 17-2 189-1 37-1 280-1 219-1 112-1 270-2 17-1 81-1 267-2 23-1 231-2 75-1
4	15-1 151-1 228-1 180-1 95-1 86-1 250-1 123-1 296-1 265-1 208-1 270-1 255-1 62-1 141-1 274-1 79-1 59-1 145-1 42-1 126-1 224-2 48-1 235-1 124-1 64-1 68-1 237-2 200-1 250-2 2-1 208-2 31-1 209-2 131-1 222-2 214-1 223-1 109-1 266-2 94-1 30-1 264-1 253-1 143-1 233-1 98-1 215-1 207-1 258-1 223-2 244-1 21-1 67-1 240-1 275-2 52-2 155-1 57-1 242-2 213-1 34-1 5-1 118-1 220-2 72-1 76-1 265-2 170-1 60-1 212-1 92-1 285-1 97-1 137-1 168-1 240-2 225-2 121-1 22-1 236-2 18-1 13-1 166-1 263-182-1
5	262-2 255-2 106-1 4-1 33-1 181-1 101-1 256-2 172-1 80-1 253-2 8-1 11-1 202-1 288-1 159-1 230-1 262-2 298-1 222-2 1-1 47-1 251-1 160-2 120-1 221-1 114-1 217-1 259-1 212-2 44-1 186-1 195-1 204-2 66-1 53-1 283-1 50-1 232-2 32-1 244-2 32-1 262-2 214-2 231-1 244-2 150-1 194-2 294-1 71-1 38-1 184-1 228-2 284-1 260-1 116-1 58-1 167-1 276-1 249-1 262-2 128-1 229-1 173-1 176-1 252-1 201-1 127-1 164-1 249-2 39-1 197-1 83-1 188-1 76-2 132-1 238-2

From Table 3, it can be seen that medium-scale orders are divided into a total of 5 batches.

From Table 4, it can be seen that large-scale orders are divided into a total of 5 batches.

### Algorithm effectiveness analysis

To validate the optimization effect of the algorithm, this section compares the order picking time, delay costs, picking costs, and the minimization of the maximum time difference before and after the improvement. The comparison results after optimization are shown in Table 5. From the table, the following points can be clearly observed:

**Table 5:** Comparison Results of the Improved NSGA-II Algorithm with Existing Algorithms.

order scale	Evaluation Metrics	Number of Employees Hired	Picking Time/s	Delay cost and picking cost/yuan	Minimize the maximum time difference/s
N = 100	Before Optimization	5	2620.5	530.6	21.3
	After Optimization		2559.8	505.2	19.5
	MOEA/D		2581.3	533.2	22.7
N = 200	Before Optimization		7184.5	3608.6	20.5
	After Optimization		6978.8	3353.2	17.7
	MOEA/D		7193.5	3670.4	19.6
N = 300	Before Optimization		11634.2	6904.7	18.6
	After Optimization		10383.6	6350.1	14.7
	MOEA/D		11349.5	7032.5	17.8

(1) As the order quantity increases, the improved NSGA-II algorithm significantly enhances the optimization of picking time. Specifically, the improved algorithm is more effective in reducing picking time for large-scale orders. Compared to the original NSGA-II, the increase in picking time is lower, indicating a clear improvement in time utilization by the optimized algorithm. This advantage becomes particularly evident when handling larger order volumes, where the improved algorithm shows a substantial benefit in controlling picking time. (2) As the number of orders increases, picking costs also rise. However, with the improved NSGA-II algorithm, the increase in picking costs is effectively controlled. The comparison results show that, for orders of the same scale, the improved algorithm reduces picking costs, especially when the order volume is large, demonstrating superior cost control. This suggests that the improved NSGA-II algorithm has a distinct advantage in reducing picking costs, providing a more cost-effective solution for practical applications. (3) As the batch size increases, the improved NSGA-II algorithm shows significant optimization in balancing the workload among pickers. Particularly when processing large-scale orders, the algorithm effectively reduces the disparity in workload between pickers, optimizing task allocation and preventing individual pickers from being overburdened. Compared to the traditional NSGA-II algorithm, the improved version better achieves a balanced distribution of workload, enhancing overall work efficiency.

**Fig 11:**
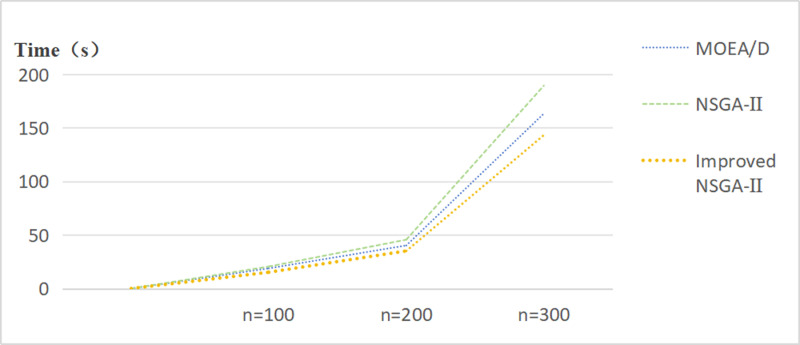
Comparison of Computational Time Chart.

The NSGA-II, MOEA/D, and Improved NSGA-II algorithms were compared in terms of their computational performance. It was observed that the running time of the Improved NSGA-II was significantly shorter than that of the traditional NSGA-II and MOEA/D algorithms. This reduction in running time can be attributed to the improvements made in the algorithm, including enhanced convergence speed, more efficient solution search, and better handling of computational complexity. The Improved NSGA-II demonstrated a clear advantage, particularly when dealing with larger problem sizes, where the reduction in processing time became more pronounced.As shown in Fig 11.

**Fig 12:**
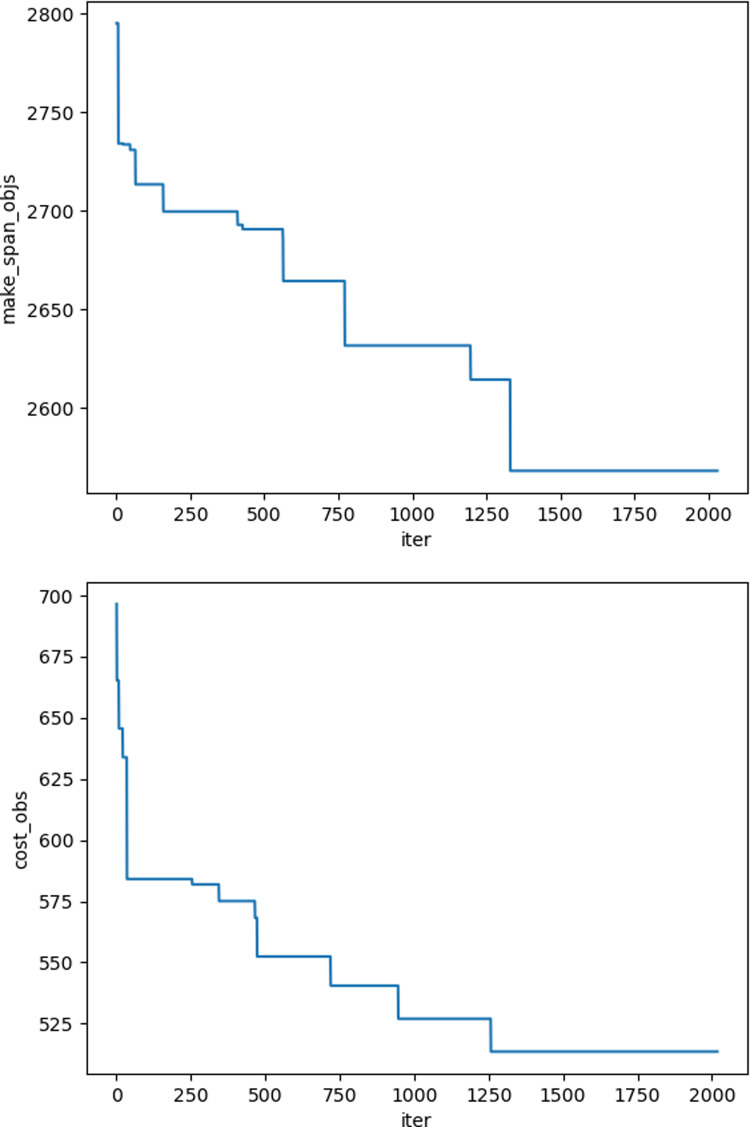
Convergence Iteration Graphs of f_1_ and f_2_ for Small-Scale Cases Based on the Improved NSGA-II Algorithm.

**Fig 13:**
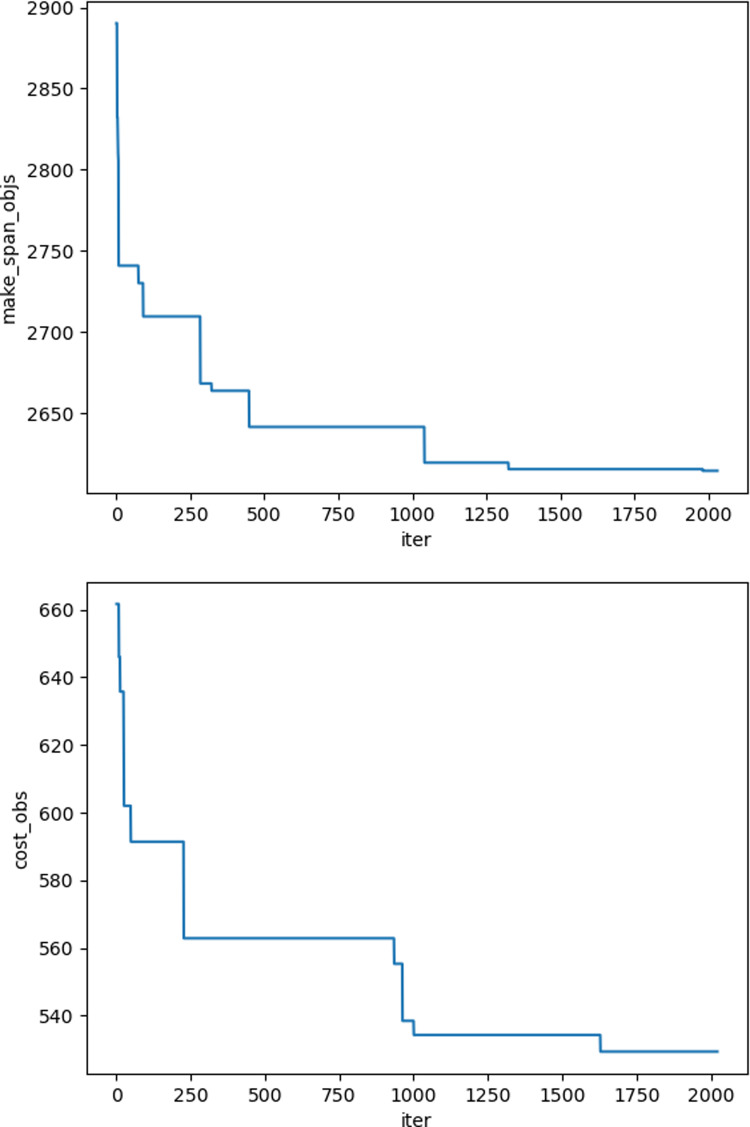
Convergence Iteration Graphs of f_1_ and f_2_ for Small-Scale Cases Based on the NSGA-II Algorithm.

**Fig 14:**
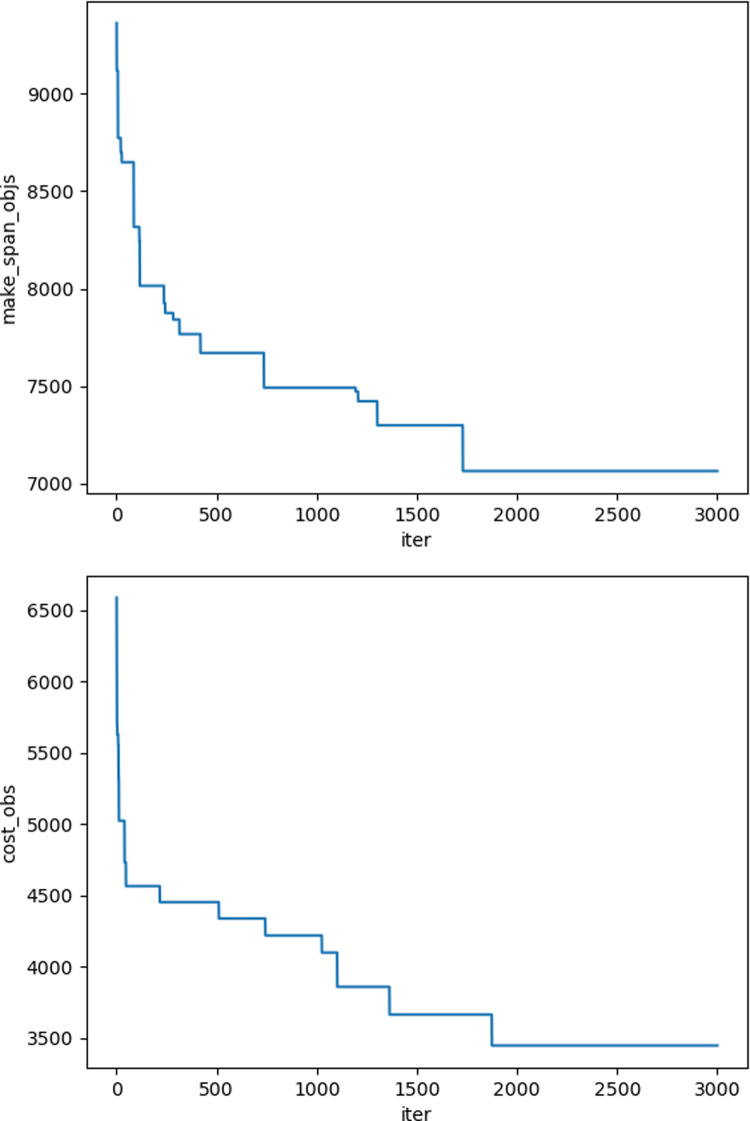
Convergence Iteration Graphs of f_1_ and f_2_ for Medium-Scale Cases Based on the Improved NSGA-II Algorithm.

**Fig 15:**
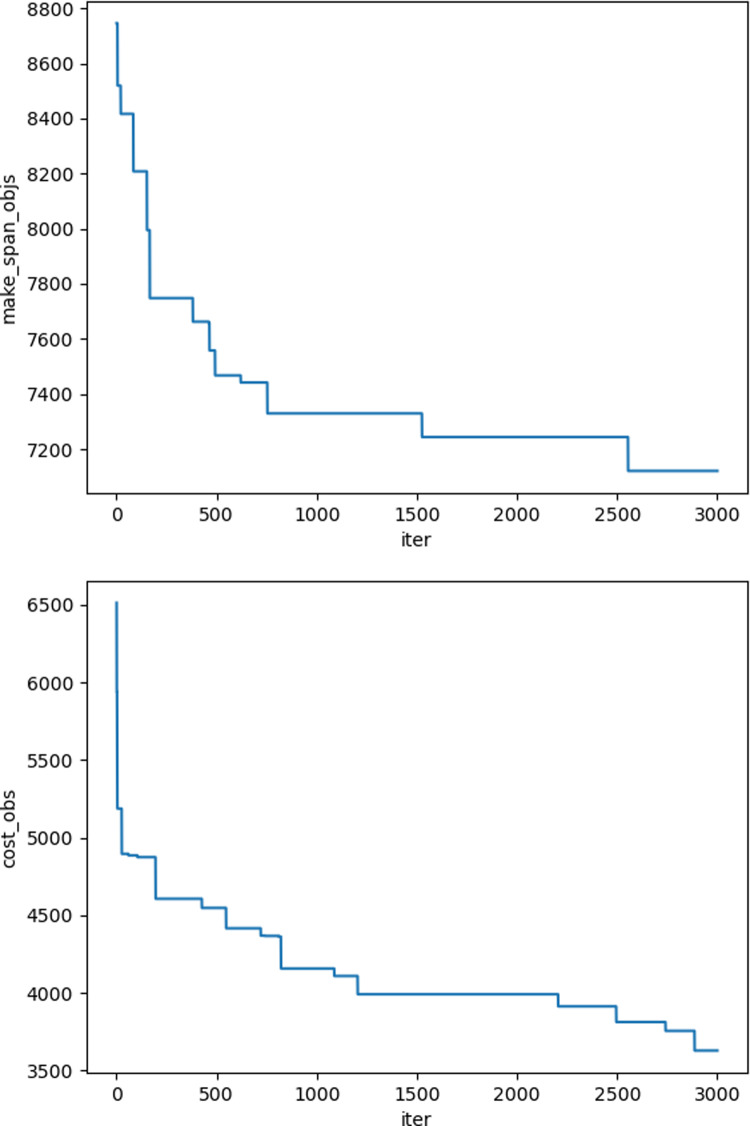
Convergence Iteration Graphs of f_1_ and f_2_ for Medium-Scale Cases Based on the NSGA-II Algorithm.

**Fig 16:**
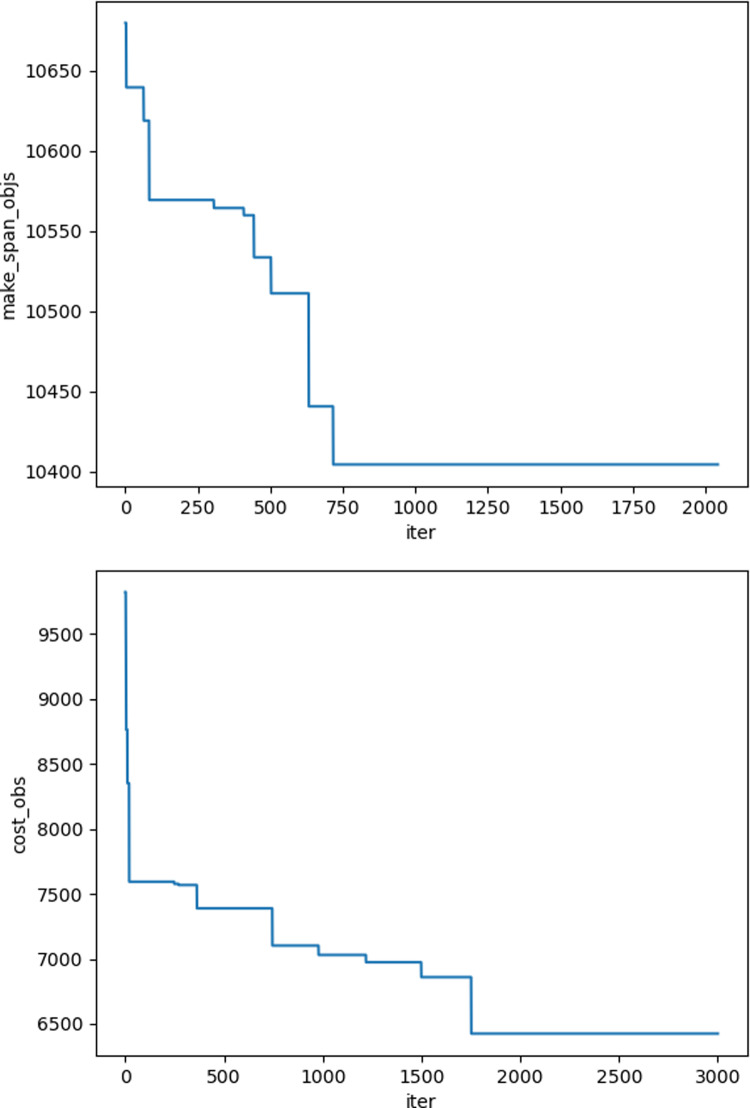
Convergence Iteration Graphs of f_1_ and f_2_ for Large-Scale Cases Based on the Improved NSGA-II Algorithm.

**Fig 17:**
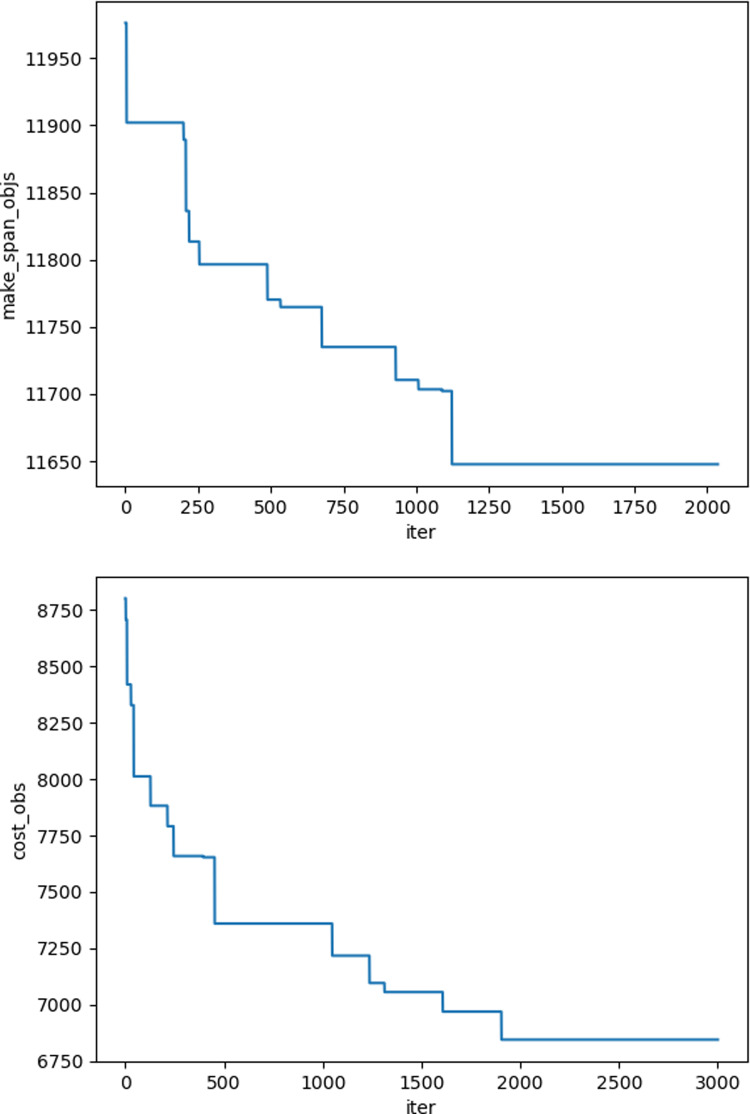
Convergence Iteration Graphs of f_1_ and f_2_ for Large-Scale Cases Based on the NSGA-II Algorithm.

The iteration graphs for the objective functions f_1_ and f_2_ based on the NSGA-II and Improved NSGA-II algorithms are shown in Figs 12–17. It can be observed that the Improved NSGA-II algorithm converges faster and yields better overall results compared to the traditional NSGA-II algorithm. Additionally, the Improved NSGA-II demonstrates higher stability throughout the optimization process. These results indicate that the proposed algorithm outperforms the NSGA-II algorithm in terms of optimization performance, demonstrating its efficiency and effectiveness in solving the problem.

Table 6 presents a comparison of the results with and without the order splitting strategy under the Improved NSGA-II algorithm. As shown in Table 6, the implementation of the splitting strategy significantly reduces both order picking time and total picking cost across different problem scales, compared to the case without the splitting strategy. Specifically, the improvement in order picking time ranges from 8.1% to 15.3%, while the improvement in total picking cost ranges from 6.7% to 18.2%. Furthermore, as the number of orders increases, the improvements become more pronounced, indicating that the splitting strategy has a more significant impact on larger-scale problems.

**Table 6:** Comparison of Order Splitting.

order scale	Order Splitting		Non-Splittable Orders	
	Picking Time/s	Delay cost and picking cost/yuan	Picking Time/s	Delay cost and picking cost/yuan
N = 100	2559.8	505.2	2767.1	539.0
N = 200	6978.8	3353.2	7816.3	3822.6
N = 300	10383.6	6350.1	11972.3	7505.8

### Sensitivity analysis

To examine the impact of the maximum cargo volume that picking equipment can accommodate, the travel speed of pickers,the speed at which picking personnel extract goods, and the cost of picking a single product from the shelf on the objective functions, a sensitivity analysis of each parameter was conducted. Each parameter value was increased or decreased by 5%, 10%, and 15% from its original value, while keeping the other parameters constant during each change. The average of the final objective values was taken, and the results are presented in Tables 7–9.

**Table 7:** The relationship between parameter variations and picking time under different scales.

order scale	Parameter Variation Range	−15%	−10%	−5%	0%	5%	10%	15%
N = 100	*V*	2449.4	2502.6	2530.8	2559.8	2607.1	2649.2	2689.4
	*v*	2762.2	2656.6	2598.3	2559.8	2503.0	2464.4	2300.1
	$v_{\text{p}}$	27107.3	2675.7	2604.4	2559.8	2409.2	2474.1	2323.2
	*c*	2556.4	2550.7	2557.2	2559.8	2556.7	2558.4	2559.9
N = 200	*V*	7110.4	7063.6	7016.8	6978.8	6923	6909.2	6893.4
	*v*	7235.2	7156.6	7058.3	6978.8	6833.0	6764.4	6700.1
	$v_{\text{p}}$	7107.3	7095.7	7024.4	6978.8	6939.2	6874.1	6803.2
	*c*	6981.4	6980.7	6977.2	6978.8	6976.7	6978.4	6979.9
N = 300	*V*	10296.4	10309.6	103472.8	10383.6	10400.2	10435.7	10460.8
	*v*	10680.8	10550.6	10474.4	10383.6	10263.8	10159.3	10067.8
	$v_{\text{p}}$	10654.9	10543.1	10489.4	10383.6	10257.8	10149.7	10092.3
	*c*	10383.3	10387.7	10380.8	10383.6	10383.9	10383.2	10382.7

**Table 8:** The Relationship Between Parameter Variations and Total Cost under Different Scales.

order scale	Parameter Variation Range	−15%	−10%	−5%	0%	5%	10%	15%
N = 100	*V*	554.2	530.7	516.7	505.2	490.1	481.9	477.0
	*v*	567.9	548.3	529.3	505.2	488.4	476.6	470.6
	$v_{\text{p}}$	594.5	576.2	532.1	505.2	457.6	408.3	380.7
	*c*	358.7	400.3	460.2	505.2	543.9	573.4	603.3
N = 200	*V*	3502.4	3451.4	3392.8	3353.2	3299.4	3203.7	3187.5
	*v*	3606.8	3571.0	3459.3	3353.2	3299.6	3172.1	3089.6
	$v_{\text{p}}$	3484.7	3408.3	3371.2	3353.2	3287.5	3210.9	3184.6
	*c*	2921.6	3076.4	3153.7	3353.2	3567.0	3779.2	3897.8
N = 300	*V*	6581.6	6487.9	6403.3	6350.1	6279.7	6207.5	6975.9
	*v*	6577.9	6501.3	6475.9	6350.1	6276.7	6223.8	6180.8
	$v_{\text{p}}$	6434.0	6399.2	6369.0	6350.1	6301.2	6285.5	6260.0
	*c*	5945.5	6134.4	6267.7	6350.1	6555.9	6781.2	6892.6

**Table 9:** The relationship between parameter variations and the balance of picker workload under different scales.

order scale	Parameter Variation Range	−15%	−10%	−5%	0%	5%	10%	15%
N = 100	*V*	18.8	18.9	19.0	19.5	19.2	19.1	19.3
	*v*	19.1	19.4	18.9	19.5	19.2	19.3	18.9
	$v_{\text{p}}$	19.9	19.2	19.3	19.5	19.7	19.5	19.2
	*c*	18.9	18.8	19.2	19.5	19.5	19.4	19.617
N = 200	*V*	17.2	17.4	17.7	17.7	17.7	17.6	17.3
	*v*	17.5	17.0	17.3	17.7	17.9	18.0	17.8
	$v_{\text{p}}$	17.4	17.6	17.7	17.7	17.3	17.8	17.7
	*c*	17.4	17.3	17.2	17.7	17.4	17.7	17.7
N = 300	*V*	14.5	14.4	14.8	14.7	14.3	14.7	14.6
	*v*	14,6	14,7	14.7	14.7	14.6	14.7	14.7
	$v_{\text{p}}$	14.5	14.6	14.7	14.7	14.7	14.8	14.7
	*c*	14.5	14.7	14.6	14.7	14.7	14.7	14.7

(1) The maximum cargo volume is negatively correlated with picking time: This indicates that the model is quite sensitive to changes in this parameter. When the picking equipment can accommodate more cargo, the time required for each picking task decreases. This is because a larger cargo capacity allows more items to be processed in a single picking operation, reducing the number of operations and transport trips, thereby shortening the picking time. Conversely, when the maximum cargo volume is smaller, picking time increases because more time and trips are required to complete each picking task. Therefore, optimizing the equipment’s cargo capacity not only improves picking efficiency but also effectively shortens the overall order processing cycle, which is particularly important in large-scale order processing. (2) The picker’s travel speed and the picking speed of the items are strongly negatively correlated with order picking time: This means the model is highly sensitive to changes in these parameters. An increase in the picker’s travel speed allows them to cover a larger area within the same period, thereby reducing picking time. Similarly, speeding up the picking of items also reduces the time required to complete each order. The impact of these two factors on picking time becomes more pronounced as the order scale increases, as pickers need to operate over a larger area with more orders. Improving travel speed and item picking speed significantly enhances operational efficiency and reduces overall picking time. Therefore, improving the travel speed and picking speed of workers is crucial for optimizing picking efficiency and reducing picking time, especially in large-scale operations. (3) The cost of extracting items has little impact on the objective function: This indicates that the model is not sensitive to changes in this parameter. Although the extraction cost may be influenced by factors such as picking speed and labor intensity, in the current model, changes in extraction costs have minimal impact on the optimization objective.

(1) Maximum cargo volume and picker travel speed are negatively correlated with cost: The maximum cargo volume and picker travel speed show a negative correlation with total cost, indicating that these two factors have a significant impact on cost changes, and the model is highly sensitive to their variations. As the order scale increases, the impact of these factors on cost becomes more pronounced. Specifically, increasing the maximum cargo volume means that the picking equipment can accommodate more items in each trip, thus reducing the number of trips and lowering the total cost. Therefore, optimizing cargo capacity is particularly important in large-scale order processing, as it can significantly improve picking efficiency and reduce operational costs. At the same time, improving the picker’s travel speed can accelerate the movement of goods during the picking process, shorten overall operation time, and reduce additional costs due to delays. Thus, enhancing picker travel speed and increasing equipment capacity both play a significant role in reducing costs. (2) Picker picking speed is weakly negatively correlated with cost: The negative correlation between picker picking speed and total cost is weak. This means that although increasing picking speed can have a positive effect on cost reduction, its impact is relatively small. Improving picking speed enables faster retrieval of items for each order, thus reducing the time and cost associated with completing individual orders. However, the reduction in cost due to speed optimization is limited, as the increase in picking speed does not significantly change the overall operation of the picking process in practice. (3) Extraction cost is strongly positively correlated with total cost: The extraction cost shows a strong positive correlation with total cost, indicating that the model is extremely sensitive to changes in this parameter. As order demand increases, the extraction cost also increases, leading to a significant rise in total cost. This is primarily because, with higher demand, pickers need to extract goods more quickly and frequently, which can lead to additional costs, such as increased labor usage, higher operational complexity, and potentially decreased system efficiency. Specifically, excessively high extraction speed may cause unnecessary bottlenecks in the system, lowering overall operational efficiency. Therefore, extraction costs need to be carefully managed, particularly in high-demand scenarios, to avoid unnecessary cost inflation from overly fast extraction processes.

From the results of Table 9, the following conclusion can be drawn: The maximization of the difference between the picking time of the most utilized picker and the average picking time of all pickers is essentially unaffected by parameter changes, indicating that the model is not sensitive to variations in this aspect.

## Conclusion

This study analyzes the impact of order batching strategies on the picking efficiency of e-commerce distribution centers in an e-commerce environment. The main objective of the research was to optimize order batching by considering three key goals: minimizing order picking time, minimizing order delay and picking cost, and achieving a balanced workload among pickers. Based on these goals, an optimization model incorporating order batching strategies was developed, with a focus on how the choice of batching strategy affects overall operational efficiency. The model aims to address the challenges faced by e-commerce distribution centers, especially in situations where large volumes of orders need to be processed efficiently and cost-effectively. The research findings provide valuable insights into how order batching decisions can significantly improve the performance of e-commerce logistics systems.

An improved NSGA-II algorithm was proposed and validated through computational examples. This algorithm aims to provide better solutions compared to traditional algorithms by enhancing convergence speed and solution quality. A series of computational experiments were conducted to evaluate the performance of the algorithm under different parameter settings. The results indicate that the proposed improved NSGA-II algorithm outperforms the conventional NSGA-II algorithm in terms of stability and reliability, providing higher-quality solutions when applied to the order batching model. Additionally, sensitivity analysis was performed to explore the relationships between parameter variations and the objective functions. This analysis identified the sensitivity of the model to specific parameters and highlighted the relationships between various parameter variables and the objective functions.

The results show that the order batching model based on the improved NSGA-II algorithm is both effective and robust. The algorithm is capable of providing high-quality solutions and demonstrates good stability, making it an effective tool for optimizing picking operations in e-commerce distribution centers. The proposed model and algorithm provide a solid theoretical foundation for improving picking efficiency and offer practical significance for enhancing operational performance in real-world applications. The successful implementation of this approach has the potential to reduce operational costs, improve delivery efficiency, and ensure a more balanced workload among pickers, which is of great value as the e-commerce industry continues to grow and order volumes increase.

## Practical applications and impacts

The order batching strategy not only improves the efficiency of the picking process but also has a profound impact on the work patterns of warehouse management practitioners. Firstly, the batching strategy demands a higher level of coordination skills and a deep familiarity with the warehouse layout from the practitioners. Secondly, the implementation of the batching strategy reduces unnecessary movement, decreases the intensity of work, and enhances job satisfaction. Furthermore, by optimizing inventory levels, it reduces stockpiling, lowers capital occupation, and thereby reduces operational costs.

## Limitations

In the model established in this paper, issues such as picking aisle congestion or product unavailability were not considered. In future research, if the aforementioned issues can be taken into account, the applicability of the research content to real-world situations would be significantly enhanced.

## Future research directions

(1) “The ‘person-to-goods’ picking model and the constancy of picking equipment are the premise of this study. In future research, the integration of higher levels of automation equipment should be considered, along with its impact on the efficiency of the ‘goods-to-person’ picking method.”
(2) There are many factors that affect the efficiency of order picking, such as warehouse layout, bin assignment. This paper only considered order batching.In future research, other factors that affect picking efficiency can be jointly considered.

## Supporting information

S1 File Data. (ZIP)

## Appendix 1: Core code

from config import Config

class Picker(object):

 def __init__(self, id, capacity, parcel_pos):

  self.id = id

  self.capacity = capacity

  self.parcel_pos = parcel_pos

  self.route = []

  self.real_route = []

  self.make_span = 0

  self.delay_cost = 0

  self.pick_cost = 0

 def reset(self):

  self.route = []

  self.real_route = []

  self.make_span = 0

  self.delay_cost = 0

  self.pick_cost = 0

 def get_pick_time(self):

  total = 0

  for task in self.route:

   total += task.process_time

  return total

 def update_route_info(self):

  cur_route = []

  cur_load = 0

  for index, task in enumerate(self.route):

   if cur_load + task.volume <= self.capacity:

    cur_route.append(task)

    cur_load += task.volume

    if index == len(self.route) - 1:

     self.real_route.append(cur_route)

   else:

    self.real_route.append(cur_route)

    cur_route = [task]

    cur_load = task.volume

    if index == len(self.route) - 1:

     self.real_route.append(cur_route)

  self.make_span = 0

  self.delay_cost = 0

  self.pick_cost = 0

  leave_last_task_time = 0

  last_pos = self.parcel_pos

  for b_id_index, tasks in enumerate(self.real_route):

   b_id = str(self.id) + "_" + str(b_id_index)

   for index, task in enumerate(tasks):

    self.pick_cost += Config.unit_pick_cost * task.quantity

    arrival_time = leave_last_task_time + last_pos.distance_to(task)

    start_process_time = max(arrival_time, task.arrival_time)

    end_process_time = start_process_time + task.process_time

    task.assign_picker_id = self.id

    task.start_process_time = start_process_time

    task.end_process_time = end_process_time

    task.assign_batch_id = b_id

    leave_last_task_time = end_process_time

    last_pos = task

   leave_last_task_time += last_pos.distance_to(self.parcel_pos)

   for index, task in enumerate(tasks):

    # 批次完成时间

    task.batch_end_process_time = leave_last_task_time

    if task.t_aw < task.batch_end_process_time < task.t_af:

     self.delay_cost += Config.alpha * (task.t_af - task.batch_end_process_time)

    elif task.batch_end_process_time > task.t_af:

     self.delay_cost += Config.beta * (task.batch_end_process_time - task.t_af)

   self.make_span += leave_last_task_time

   last_pos = self.parcel_pos
