# Correction: Research on order batching optimization based on improved NSGA-II algorithm

**DOI:** 10.1371/journal.pone.0331708

**Published:** 2025-09-04

**Authors:** Huiyue Xu, Juping Shao, Yanan Sun

The first author, Huiyue Xu, is incorrectly noted as the corresponding author. The correct corresponding author is Juping Shao. Juping Shao’s email address is: wlustbshao@163.com.
